# Publication Ethics: Many Facets, Collaboration Required

**Published:** 2013-05

**Authors:** Viroj Wiwanitkit

**Affiliations:** 1 Visiting Professor, Hainan Medical University, China; Adjunct Professor, Joseph Ayobabalola University, Nigeria; 2 Visiting Professor, Faculty of Medicine, University of Nis, Serbia

Sir, the recent publication on “Publication Ethics” is very interesting (1). The article by Fazly Bazzaz and Sadeghi demonstrated many interesting cases of misconducts. Indeed, the misconducts can be seen in many ways. As mentioned in the present publication, both author and editor can perform publication misconducts. Nevertheless, the problem has many more facets. Sometimes, the misconducts are generated by the third parties. For example, the publisher can perform publication misconduct. In fact, the editor takes the role in control of the materials to be published in the journal. However, the publisher sometimes interferes with the process. Some unethical publication can be seen in some “predator” online publisher. “Pay then publish” without control of its quality can be seen (see also http://www.nature.com/news/predatory-publishers-are-corrupting-open-access-1.11385 and scholarlyoa.com/2012/12/06/bealls-list-of-edatory-publishers-2013). In the present era of rapidly increased number of open access journal, this problem is expected to increase. Another example is the criminal case of “one disguising to be the other person” to perform misconduct aiming at discrediting or destroying others. An interesting case was previously published in Hepatitis Monthly; an author performed plagiarized articles behind the name of another person and finally was charged by the IT investigation (2). Finally, the institute sometimes also performs misconduct. This can be supporting of their members who perform scientific misconduct by acts which do not correspond to the problem (this problem is usually seen in the problematic case generated by senior faculties or administrators), dealing with publisher to “buy” supplementary volume for publication of the works from the institute with low standard of peer reviewing process, citing the name of some famous scientists into the publication of the institute without previous asking for permission, etc. In order to cope with those problems, the collaboration between all partners (author, editor, journal, institute, reviewer and reader) is needed. Sharing of knowledge, learning together and going together toward the ethical publication is recommended.
